# Mechanisms and Efficacy of Massage Therapy for Post-Exercise Muscle Repair: A Narrative Review

**DOI:** 10.3390/muscles5020029

**Published:** 2026-04-21

**Authors:** Peter M. Tiidus

**Affiliations:** Department of Kinesiology, Faculty of Applied Health Sciences, Brock University, St. Catharines, ON L2S 3A1, Canada; ptiidus@brocku.ca

**Keywords:** massage, muscle repair, inflammation, edema, protein synthesis, muscle force recovery, massage in humans, massage and animal models, mechanotransduction, muscle satellite cells

## Abstract

Although widely used, massage has not been reported to be effective in enhancing recovery from exercise-induced muscle damage in humans. Studies using massage-like interventions in animal models have, in contrast, consistently demonstrated a significant enhancement of muscle repair, reduction in muscle inflammation and enhanced return of muscle force following muscle damage. The physiology of muscle damage and repair and the putative physiological mechanisms of potential massage-induced muscle repair and post-damage recovery, including soreness sensation, edema, inflammation, protein synthesis and other related mechanisms, are reviewed in this context. Animal models have demonstrated that massage effectiveness in enhancing post-damage muscle repair is dictated by the timing, duration, force and technique of its application and may also be modified by age and sex effects. The potentially very narrow “window of effectiveness” of massage application for the enhancement of post-damage muscle repair in humans has yet to be defined. And the lack of demonstrated effectiveness for massage on post-damage muscle recovery may be due to the wide range and inconsistency of massage techniques, timing and methodologies applied in human studies. Until a specific massage application protocol is defined for massage efficacy in post-damage human muscle recovery, therapists will continue to work blind, using a variety of techniques which lack empirical validity and have an undemonstrated effectiveness for enhancing muscle repair.

## 1. Introduction

Massage therapy is widely used to enhance skeletal muscle repair and recovery following exercise- or overuse-induced muscle damage. Ongoing research has begun to uncover the physiological mechanisms by which massage may achieve any potential benefits for muscle healing, as well as the limitations of current massage therapy practices to enhance muscle repair in humans. This narrative review will cover the putative physiological mechanisms for the potential effectiveness of massage and tissue-manipulative therapy in various forms to enhance muscle repair following damage and the evidence for its effectiveness, as well as the engagement of these physiological mechanisms associated with muscle repair and regeneration processes by massage. Relevant animal and human studies are included. Specifically, the effectiveness of massage in engaging the following putative positive physiological effects associated with muscle repair will be critically reviewed: enhanced muscle blood flow, oxygenation and removal of metabolic byproducts; the amelioration of muscle soreness, swelling and edema; the influence on muscle stiffness; the optimization of muscle and systemic inflammatory responses; the optimization of muscle repair and regeneration signaling pathways; changes to rates of muscle protein synthesis and degradation and muscle satellite cell activation.

Not all of the above potential mechanisms for the effectiveness of massage are supported by research. Nevertheless, there is growing evidence, primarily from animal models, that massage can enhance repair following muscle damage via a number of the above mechanisms and that the timing, length and pressure of the application of massage may be significant factors in its efficacy. More research is required to validate and define the effectiveness of massage and its specific techniques on influencing muscle repair mechanisms, particularly in humans.

## 2. Massage and Tissue-Manipulative Techniques

Manual massage as performed by a human massage therapist tends to consist of several inter-related techniques as described by Goats [1]. Effleurage consists of stroking movements from proximal to distal, parallel to the long axis of tissue which compresses tissue. Petrissage involves the forceful compression, twisting and rolling of tissue. Friction involves penetrating transverse pressure applied by the fingertips. Tapotement involves the percussive clapping or pounding of tissues to enhance vibratory effects and the vibration or shaking of tissue, with the hands firmly in contact with the skin.

Other equipment-related techniques include foam roller or stick massage [2,3], which involves pressure applied in a rolling motion over muscles and limbs using a rolling device, and hydromassage or underwater jets, as in a whirlpool applied to muscles [1]. The application of massage in humans also involves a variety of intensities, durations and timings of massage applications which have not been standardized based on empirical evidence of effectiveness.

Experiments in rodent and rabbit models which mimic massage have often involved some forms of mechanical rolling, which induces cyclical compression of a fixed load on muscles, or the specific physical application of direct massage [4,5,6]. Animal-based studies have generally better defined and standardized the range of massage techniques, durations, force and timings than those involving humans.

For the purposes of this review, the various forms of tissue manipulation involving massage or massage mimetics will be collectively referred to as massage. Specific manipulative techniques will be noted where appropriate.

## 3. Physiology of Muscle Damage and Repair

To appreciate the potential for massage to influence muscle damage and repair and related manifestations following exercise-induced injury, the physiology of muscle damage and repair mechanisms will be very briefly reviewed. The following summary is based on several well-documented reviews [7,8,9,10].

Initial muscle damage resulting from overuse and, particularly, eccentric muscular contractions will manifest as micro-tears in the muscle sarcolemma and muscle sarcomere, z-line disruption, and the degradation of structural proteins such as dystrophin and desmin. The leakage of muscle cytoplasm and cytoplasmic proteins such as creatine kinase into the blood consequent to sarcolemma injury-induced permeability will also take place. The initial damage is followed by an inflammatory response, with pro-inflammatory and anti-inflammatory cytokines and other signaling ofinitial neutrophil and a subsequent macrophage infiltration of the muscle, which then serves to help break down and remove damaged muscle tissue, initiate muscle necrosis, and ultimately signal for the initiation of repair processes. The sensation of muscle soreness and tightness or stiffness that often accompanies exercise-related muscle damage can result from inflammatory- and edema-related factors, as well as muscle fascia or neuromuscular reactions to soreness and disruption. The further signaling and control of inflammatory responses from a wide range of inputs regulate the inflammatory and subsequent repair processes, which involve muscle satellite cell activation and proliferation to repair and replace damaged muscle fibers and regulate and signal a net-positive protein synthesis and degradation balance. There is a general loss of muscle force capacity resulting primarily from damage to sarcomeres and sarcolemma, which gradually returns to normal at timelines similar to inflammation and repair processes.

While exercise and overuse will generally induce modest muscle injury and soreness sensation, contusion and more severe muscle strain can cause significantly greater damage, which can also result in the formation of connective scar tissue in muscles during the repair process.

## 4. Does Massage Enhance Muscle Blood Flow, Oxygenation and Removal of Lactic Acid?

This section reviews evidence demonstrating how muscle blood flow and potential related effects are not influenced by massage in humans.

### 4.1. Blood Flow

One of the earliest and still occasionally cited potential mechanisms by which massage therapy has been reported to act has been its possible positive effects on muscle blood flow [11,12]. Enhanced muscle blood flow would potentially increase oxygen delivery, the removal of metabolic byproducts, potentially ameliorate muscle swelling and edema and enhance the arrival of neutrophils and macrophages related to post-damage inflammation and muscle repair [13]. Any or all of these effects could potentially contribute to enhanced muscle recovery by massage therapy. For example, significantly enhanced muscle oxygenation, as demonstrated by hyperbaric oxygen treatment, will positively affect muscle repair, particularly following traumatic injury [13]

Convincing evidence using Doppler ultrasound recordings of blood flow in humans has repeatedly demonstrated that various forms of massage will not enhance arterial flow into muscles [14,15,16,17] or venous outflow from muscles [14] and may even impair muscle arterial blood flow following exercise [15,17]. For example, Shoemaker et al. [15] demonstrated that three common types of massage used on both large (thigh) and small (forearm) muscle groups will not increase the net muscle blood flow as measured in the femoral and brachial arteries, respectively. They demonstrated that, if muscle blood flow was effective in enhancing muscle healing, modest muscle contractions would induce significantly more muscle blood flow than massage and would thus be far more effective in enhancing muscle healing via blood flow than massage.

There is evidence that skin and peripheral tissue microcirculatory blood flow can be enhanced by manual and foam roller massage in both the massaged and contralateral limbs [16,18,19]. Nevertheless, without enhanced overall arterial blood flow to the limb, enhancing skin blood flow in massaged limbs via massage may actually decrease the overall blood flow and oxygenation to the muscle itself, thereby diminishing any potential blood flow-related benefits [16]. Hence, as has been demonstrated, muscle blood flow is not influenced by massage and cannot be a factor in any potential benefits of massage on post-exercise muscle recovery or repair.

### 4.2. Lactic Acid

The presence of lactic acid in the muscle has, at least in the mind of the general public, been associated with post-exercise muscle soreness and muscle damage [20]. However, it is well known that lactic acid is cleared from muscles within minutes following intense exercise, and muscle soreness does not peak until at least 24–48 h post-exercise [21]. Eccentric muscle contractions induce significant muscle damage and muscle soreness, with little lactic acid accumulation, while concentric muscle contractions result in much less muscle damage and muscle soreness, while producing more lactic acid [21]. These disconnects with muscle lactic acid and soreness sensation clearly demonstrate that lactic acid cannot be a cause of or be associated with muscle soreness. Nor can lactic acid accumulation be a cause of or a mechanism associated with muscle damage or its removal associated with muscle repair [20]. Furthermore, it has been demonstrated that massage will not enhance lactic acid removal from skeletal muscle, from systemic circulation [22], or from muscles [17]. Thus, the idea that lactic acid can have any influence on muscle damage, repair or soreness sensation or that massage can influence the removal of lactic acid from systemic circulation or muscles and thereby influence muscle soreness sensation or muscle repair can be entirely dismissed.

## 5. Does Massage Reduce Post-Exercise Muscle Stiffness?

This section describes and defines the potential effects of massage on muscle stiffness, primarily with evidence primarily from human studies.

### 5.1. Physiology of Post-Exercise Muscle Stiffness

Post-exercise muscle stiffness is thought to be related to acute alterations in the connective tissue or fascia surrounding muscle and muscle edema [1,23,24]. It has been suggested that factors associated with muscle damage may increase tissue viscosity by reducing the viscosity of hyaluronic acid, which facilitates gliding between muscle and muscle fascia, thereby reducing the flexibility of muscles and increasing stiffness [2,3,23]. Massage in various forms may potentially increase hyaluronic acid mobility or viscosity via pressure or muscle temperature changes and positively affect muscle tendon compliance [2,25,26], thereby potentially reducing post-exercise muscle stiffness.

Myofascial trigger points have also been implicated in muscle stiffness and associated pain sensation [1,27]. Trigger points are painful locations in muscles that may be caused by dehydration or injury-related fascial adhesion, resulting in a reduction in elasticity and pain [2,27], or by spinal nerve involvement outside the muscle [27]. Myofascial trigger point identification and treatment have not been well documented but could potentially respond to massage-related pressure. Ameliorating trigger point activity by various forms of massage could potentially result in reduced pain sensation and muscle stiffness [2]. However, the documentation of such specific effects of massage on muscle trigger points, as well as their direct effects on muscle stiffness, is lacking.

Since no changes in muscle electrical activity as determined by electromyography (EMG) are seen following eccentric exercise-induced muscle damage, changes in direct neural activity are not thought to contribute to muscle stiffness post-exercise [23,25].

### 5.2. Effects of Massage on Muscle Stiffness

Although findings have not been entirely consistent [23], the post-exercise determination of muscle stiffness using myotonometry [28], range of motion [23,29], and EMG [23] analysis following various types of massage in humans have generally found little or no effect of massage on these outward measures of muscle stiffness. Small positive changes in muscle flexibility have been noted immediately following massage in undamaged muscles, but these effects are transitory [30].

A double-blind study by Kong et al. [28] followed massage effects on muscle stiffness as determined by mytomometry. Myotometry is a validated measure of muscle stiffness which elicits dampening oscillations of the muscle as the muscle stiffness indicator [31]. Massage or a placebo inactive ultrasound was applied immediately following downhill running to several leg muscle groups, and while muscle stiffness increased over the next 96 h in all muscles studied, massage had no effect on the time course or degree of muscle stiffness [28]. Six weeks of massage combined with other treatments was also reported to have only minimal effects on muscle trigger point activity in subjects experiencing chronic muscle pain [32].

However, a comprehensive review of effects of foam roller massage in humans concluded that the preponderance of evidence supported a decrease in muscle stiffness and improvements in range of motion following exercise-induced muscle damage attributable to foam roller massage, which could be related to the above changes in muscle facia and muscle trigger point responses as well as possible parasympathetic neural effects and a reduction in global pain sensation facilitating range of motion benefits [2].

Nevertheless, not all studies find positive results with roller massage, with a recent study of repeated roller massage over several days not showing positive effects on muscle stiffness, as determined by limb range of motion measures [3]. A more recent study [33] also reported no effect of immediate or 48 h post-exercise massage application on muscle stiffness, as determined by ultrasound elastiography.

While more comprehensive studies are still needed, the current evidence is inconsistent and does not strongly support the ability of manual or roller massage to significantly influence muscle stiffness or the possible physiological factors associated with muscle stiffness following muscle-damaging exercise. Changes in muscle stiffness are only one of many factors associated with muscle damage and recovery.

## 6. Does Massage Ameliorate Muscle Soreness by Diminishing Swelling, Edema and Related Inflammation?

This section reviews evidence from human studies for the effectiveness of massage in diminishing muscle soreness and related manifestations from both human and animal studies.

### 6.1. Muscle Damage and Muscle Soreness

Muscle soreness often accompanies exercise-induced muscle damage, with sensations tending to peak about 48 h after damaging exercise and subsequently subsiding over the next several days [20,34]. Since the soreness sensation does not manifest until some 8–24 h following exercise, it is often referred to as “Delayed Onset Muscle Soreness” or DOMS. DOMS may be related to factors associated with the post-damage inflammatory response, including increased pressure in muscles from edema buildup [34,35] and the accumulation of inflammation-related chemicals such as histamines, bradykinins, prostaglandins and other substances released during muscle damage and inflammation which build over this time period [36]. These substances, as well as increased intra-muscular pressure, can be sensed by afferent nerve endings located in the perimysium surrounding muscle cells near the blood-borne entry for leucocytes that can generate inflammatory responses [37].

DOMS itself may not be directly related or proportional to exercise-induced muscle damage and cannot be used as a direct measure of the extent of muscle disruption or the rate of muscle repair [35]. DOMS is often greatest following an initial unaccustomed exercise bout and will, as a result of rapid muscle infrastructural remodeling and neural adaptations, be significantly ameliorated following a subsequent, post-recovery exercise bout [38,39]. The degree of exercise-induced damage to muscle sarcomeres and contractile proteins does not correlate well with DOMS sensation in different conditions [20] and cannot necessarily be used to directly assess recovery from muscle damage.

Although results vary considerably with the study design, application of massage technique, timing, and type of exercise used to induce muscle damage, there is an accumulation of evidence suggesting that massage in humans may have overall modest effects in reducing DOMS either temporarily, immediately following massage or as a longer-term amelioration [25,30,34,35,36,40]. While not all studies report benefits of massage on DOMS sensation (e.g., [3,41]), meta-analyses, comprehensive reviews [30,34,35], and controlled studies in younger and older humans [25,40,42] on the effects of single and/or multiple bouts of various types of massage conclude that the overall evidence favors modest reductions in DOMS sensations after muscle-damaging exercise, with greater reductions at 48 and 72 h post-exercise than at 24 h post-exercise [25,36,40]. Can this modest possible benefit be related to the ability of massage to influence muscle edema or inflammation following muscle damage? Or are there other possible systemic or pain-dampening effects that massage might invoke to reduce post-exercise pain sensation [2,40]?

### 6.2. Edema

Muscle damage is associated with a significant increase in muscle swelling and edema in the muscle compartment and interstitial spaces [43]. If massage can reduce muscle edema and resultant intramuscular pressure, this may be a factor in diminishing the DOMS sensation related to muscle damage. Massage has often been promoted as having potential benefits in reducing muscle edema and swelling as a means of amelioration of muscle soreness [20,44].

There is relatively little direct evidence for an effect of massage on muscle edema following exercise-induced muscle damage in humans. While massage has been shown to be effective in enhancing lymph flow and reducing subcutaneous edema in humans [45,46], there is little research on its ability to directly affect muscle edema. The determination of massage’s effect on muscle swelling and edema using limb circumference as a crude indirect measure has produced mixed results [3,29]. However, a study by Nunes et al. [33] using ultrasound echo intensity as a more direct measure of edema in vastus lateralis muscle to assess the effects of massage on post-exercise muscle swelling and edema found no effects of immediate and 48 h post-exercise massage.

Animal models have supported the potential effectiveness of massage on enhancing overall lymph flow and in reducing post-exercise muscle edema [47,48]. Lima et al. [48] cited several animal studies which used tracers to determine lymph flow and demonstrated a significant lymph flow enhancement with massage application. Hence, while massage in animal models demonstrated enhanced lymph flow and potential for the amelioration of muscle edema, this effect has yet to be conclusively demonstrated in humans.

### 6.3. Inflammation

Unlike muscle blood flow and edema, muscle damage-induced inflammatory responses and signaling have been demonstrated to positively respond to massage interventions in animal and human models [4,37,49,50,51]. These effects will be discussed in detail in the subsequent section. A very limited number of studies examined the effects of massage on some systemic markers of post-muscle damage and inflammation concurrent with its effects on DOMS [51,52]. Despite the amelioration of blood levels of various inflammatory signaling factors including Interleukin-10 (IL-10), Interleukin-8 (IL-8) and Tumor Necrosis Factor-alpha (TNF-a) following massage performed immediately and 1, 2 and 24 h after muscle-damaging exercise, White et al. [52] found no effect of massage on the concurrently measured DOMS sensation in humans. An earlier human study did find modest reductions in circulating neutrophils (up to 8 h post-exercise) and slightly decreased DOMS (up to 96 h post-exercise) when massage was performed shortly after exercise relative to a control group [52].

As previously noted, research has tended to demonstrate modest positive effects of massage on muscle soreness sensation; while studies have repeatedly shown positive effects of massage on various measures of inflammatory response in humans, causal effects of inflammation on DOMS sensation have yet to be directly demonstrated [49,51]. Thus, as will be further detailed in the following section, massage will have ameliorating effects on various aspects of post-exercise muscle and circulating inflammatory responses, but the relationship of massage-related amelioration of inflammation to any consequent diminishing muscle soreness sensation is yet to be firmly established.

Type II and IV afferent nerves located in the muscle perimysium respond to noxious stimuli including chemicals such as histamines, bradykinins and prostaglandins released with muscle damage and the subsequent infiltration of muscles by neutrophils and macrophages [37], likely contributing to the development of DOMS sensation. While these and similar changes associated with acute muscle damage may contribute to pain and DOMS sensation [53], the effects of massage on these types of stimuli in muscles are not well documented.

However, at least one rodent study that employed a post-exercise massage-like manipulation of exercised limbs reported decreased discomfort-related behaviors and ameliorated afferent nerve signaling in the massage-like treatment groups [54]. Since massage does appear to diminish muscle and systemic pro-inflammatory responses and enhance muscle recovery in animal models [4,55], even if a direct relationship has yet to be demonstrated, it is possible that some of the effects of massage on DOMS sensation may be attributable to the resultant changes in pain-inducing chemicals associated with inflammation and resultant reduction in afferent nerve pain-sensing activity in humans.

### 6.4. Other Potential Mechanisms for Effects of Massage on Muscle Pain and Soreness

In addition to DOMS, the treatment of chronic muscle pain with massage is common [1,3,11]. DOMS and chronic muscle pain may have a similar etiology [40] and hence respond similarly to massage intervention. However, there are limited and contradictory findings as to the benefits of massage for longer-term pain reduction, with two comprehensive reviews finding little consistent evidence to support positive effects of massage on chronic neck and lower back pain in humans [56,57].

In a comprehensive review of roller massage in humans, Behm and Wilkie [2] suggested that, since several studies had demonstrated post-exercise pain reduction in muscles from both roller-massaged and non-massaged limbs, global, neural gate control mechanisms may also contribute to the pain reduction effects of massage. Diffuse noxious inhibitory control (DNIC) involves activation of the type III and IV muscle afferent nerves by massage-induced pressure signaling, which can then induce the efferent neural stimulation of opioid receptors that can globally inhibit pain sensation [2,58]. Law et al. [40] have also suggested, based on rodent models, that massage-induced pain inhibition via efferent nerve activation of central mechanisms may also invoke oxytocin release. Hence, if massage is effective in reducing acute DOMS or chronic muscle pain, it may at least in part be due to its ability to invoke acute central nervous system responses that can induce modest global analgesia via DNIC-activated pathways [2,40]. Further research is required to examine the viability of this hypothesis.

## 7. Does Massage Ameliorate Muscle and Systemic Inflammatory Responses?

This section discusses the effects of massage on inflammation related responses from both animal and human studies.

### 7.1. Physiology of Post-Damage Muscle and Systemic Inflammation

Post-exercise muscle inflammation and inflammatory signaling responses are triggered by muscle damage and influence and regulate subsequent muscle repair and regeneration-related mechanisms [8]. To be most effective, an optimum inflammatory response is needed to initiate the removal of damaged muscle tissue and to initiate muscle repair. Too much inflammation, including the infiltration of muscles by leukocytes such as neutrophils and pro-inflammatory macrophages, exacerbates damage and too little results in diminished or delayed muscle repair [8]. Neutrophils, while important in helping remove damaged muscle tissue, can also exacerbate muscle damage, while various types of macrophages can participate in both inflammation-related muscle disruption as well as in anti-inflammatory signaling and the initiation of muscle repair mechanisms [8]. The potential effects of massage on muscle pro- and anti-inflammatory factors could therefore be important pathways for enhancing muscle repair and recovery following muscle damage.

Inflammation follows exercise-induced muscle damage, with increases in systemic and muscle levels of inflammatory mediators. These include pro-inflammatory cytokines such as interleukin (IL) IL-1, IL-6, IL-10 and Tumor Necrosis Factor-alpha (TNF-α) and anti-inflammatory mediators including IL-4 and Transforming Growth Factor-beta (TGF-β), arising from, and in conjunction with, the infiltration and activity of various leukocytes including neutrophils, monocytes and macrophages [8]. As noted in the previous section, massage has been demonstrated to have positive ameliorating effects on various muscle and systemic inflammatory signals and responses.

### 7.2. Massage and Inflammatory Responses

The last decade or more of research using rabbit and rodent models with controlled and quantifiable massage-like compressive loading (MLL) applications or direct massage manipulation has demonstrated significant effects of massage on post-damage muscle inflammation and subsequent muscle recovery and repair (the latter will be discussed in more detail in the subsequent section) [4,5,37,55,59,60]. Massage may affect various post-damage inflammatory responses via signaling initiated by pressure or “mechanotransduction” effects on the muscle [37]. Mechanotransduction is the “transformation of a mechanical stimulus into a chemical signal or the resulting cellular signaling cascade after an external mechanical deformation of tissue” [37]. Massage-induced pressure may directly induce changes in anabolic and immunomodulation signaling pathways in muscle, which will directly influence post-damage inflammatory responses such as muscle neutrophil infiltration and the removal of damaged tissue and transformation of macrophages from pro- to anti-inflammatory and repair signaling action [4].

Butterfield et al. [47] reported that 30 min of massage-like roller-induced mechanotherapy (MLL) applied to exercise-injured rabbit muscles significantly reduced the infiltration of inflammatory neutrophils and macrophages. This model has repeatedly demonstrated reductions in various post-exercise muscle inflammatory responses in different applications and ages of animals [4].

Barbe et al. [5] employed 10 min of manual therapy three times per week for three weeks on rodents’ upper limbs injured with repetitive movement tasks. This massage-like therapy resulted in decreased damage-induced infiltration of pro-inflammatory CD68+ macrophages and decreased the presence of pro-inflammatory cytokines IL-1b and IL-10 in muscles and tendons.

A more severe model of muscle damage induced by snake venom injection and ischemia in mice also demonstrated a significant reduction in muscle neutrophil infiltration and enhanced muscle recovery when exposed to a mechanically induced simulation of muscle massage [61]. Cezar et al. [60] also mimicked massage-like manipulation in mouse muscles following damaging toxin injection and reported a reduced infiltration of neutrophils compared to unmassaged controls. Barbe et al. [4] found significant reductions in post-damage neutrophil infiltration following massage mimicking the controlled compression of rodent muscles, which also facilitated quicker muscle repair and a reduction in fibrosis. Using the “Chinese Tuina” method, which applies direct pressure to muscle areas similar to trigger points, Lui et al. [62] also reported reduced muscle inflammatory markers and enhanced indices of muscle repair following contusion-induced muscle injury in rats.

Citing studies by Metzler et al. [63,64], Lowery et al. [50] described in vitro muscle culture studies which simulated exercise-induced injury to muscles and stretch-like interventions simulating massage-like muscle manipulations. These studies also reported reductions in the production of pro-inflammatory cytokines IL-6 and IL-8 in injury-simulated muscle cultures when exposed to massage-like manipulations.

Massage has also been effective in reducing indices of inflammatory responses in muscle and blood in humans [49,51,52]. Crane et al. [49] applied 10 min of massage to quadricep muscles immediately following exercise and reported reduced levels of pro-inflammatory cytokines IL-6 and TNF-a in the muscles. White et al. [51] also found a faster return to normal levels of the pro-inflammatory cytokine TNF-a but a delay in return to normal levels of IL-6 in blood with massage following high-intensity exercise in humans. Smith et al. [52] also reported reduced levels of circulating neutrophils up to 10 h post-exercise when massage was applied within two hours after muscle-damaging exercise in humans. These results collectively point to a positive effect of massage in managing post-damage, muscle and circulatory inflammatory responses, particularly in limiting post-damage muscle neutrophil infiltration in animal, in vitro, and human studies, which can contribute to reduced post-exercise damage and enhanced muscle recovery following muscle-damaging exercise.

Overall, there appears to be consistent support for the amelioration of post-damage muscle leukocyte infiltration, primarily from animal models. The evidence for systemic changes in circulating inflammatory mediators such as IL-6 and TNR-a in humans is inconsistent and requires further research clarification.

### 7.3. Timing, Pressure and Frequency of Massage Effects on Muscle Inflammation

While, in general, evidence points to the positive effects of massage on muscle inflammatory responses, the effectiveness of massage in animal models is influenced by the timing, frequency and force of the massage interventions. Moderate-force MLL for 30 min on muscles performed immediately after eccentric exercise and repeated for four days in rabbits was much more effective in diminishing muscle infiltration by neutrophils and macrophages than when MLL application was delayed by 48 h [55]. If massage is not initiated immediately following exercise-induced muscle damage in animal models, subsequent applications of massage are significantly less effective in diminishing muscle inflammatory responses and, as will be discussed in the next section, muscle recovery, regardless of their frequency [4,5].

The amount of pressure used with MLL was also critical in optimizing post-exercise muscle inflammatory responses and subsequent muscle recovery, with too little pressure having little effect and too much pressure resulting in further damage and hyper-inflammatory-related responses in animal models [37,59,61]. These factors, including massage pressure, technique, duration and timing, which have been well controlled and documented in animal models, are not nearly as well controlled or documented in massage studies involving humans and, as will be discussed, may be related to the heterogeneity of results and the difference in findings of massage benefits between animal- and human-based studies [30,48].

## 8. Does Massage Enhance Muscle Repair and Recovery?

This section discusses evidence for the effectiveness of massage in enhancing muscle recovery and repair and contrasts findings between animal and human models.

### 8.1. Physiological Mechanisms for Massage Effects on Muscle Recovery and Repair

Following exercise-induced muscle damage, muscle recovery will occur over the subsequent 7–10 days. This recovery will involve the removal of damaged tissue and initiation of muscle repair and rebuilding involving muscle satellite cell activation, ribosomal proliferation, and enhanced muscle protein synthesis and turnover, all contributing to muscle repair, strengthening and remodeling [9]. The activation and proliferation of muscle satellite cells is required for muscle repair and regeneration to take place [65]. As with muscle inflammatory responses, muscle recovery and repair has been shown to be significantly enhanced by massage-like interventions, particularly in animal and in vitro models [4,5,37,66].

As described in the previous section on inflammation, direct pressure on muscle or mechanotransduction can, possibly via the extracellular matrix (ECM), activate signaling pathways related to immune responses, as well as direct anabolic signaling in muscles, that promote the process in muscle noted above that enhances muscle recovery and repair following muscle damage and reduces post-damage muscle fibrosis accumulation [4,5,60,61,66,67]. The ECM is linked to muscle contractile protein, actin, via integrin proteins which sense mechanotransduction and help reduce muscle damage via binding to muscle basement membrane laminin through integrin regulation [67]. While signaling cascades for the mechanotransduction effects of massage have been demonstrated in vitro, the influence of these pathways on massage-induced effects on muscle repair await further experimental confirmation in both animals and humans [67].

Muscular contractions, as occur during exercise such as resistance training, also place strain on and stretch muscle membranes and contractile infrastructure. These muscle strains are sensed and translated into protein kinase-regulated signals for muscle hypertrophy and protein synthesis, such as the insulin-like growth factor-1 (IGF-1) pathway, which signal further downstream regulatory factors such as protein kinase B (AKT) and mechanistic target of rapamycin (mTor) [68,69]. Exercise strain on muscles also induces signaling factors such as IGF-1 and Transforming Growth Factor-beta (TGF-b) among others, which regulate the activation, proliferation, and differentiation of muscle satellite cells that will act to replace damaged muscle fibers [9,61].

The previously noted repeat bout effect which reduces muscle damage during a second bout of exercise following recovery from a previous bout is also, in part, mediated by remodeling of the ECM [67,70,71]. It is possible that specific degrees, timing and duration of direct pressure applied during massage and massage-like applications to muscle may invoke similar pathways and muscle protein synthesis responses as those associated with resistance exercise and thereby may, within a defined window of the right conditions, augment muscle repair and recovery [4]. These mechanisms for massage effects on muscle repair, while possible, await additional experimental confirmation.

### 8.2. Evidence for Enhanced Muscle Repair and Recovery Following Massage-Like Interventions in Animal Models

In a review of their work on massage effects in animal models, Van Pelt et al. [4] summarized the effects of MLL on post-exercise muscle repair and recovery. Earlier studies by this group [47,55] examined the effects of MLL on post-eccentric exercise muscle recovery in rabbit muscles. They found that muscles massaged for 30 min with MLL immediately and daily for four subsequent days had significantly less myofibrillar disruption and inflammation and much quicker and more complete repair of damage, remodeling and recovery than the unmassaged muscles. Importantly, functional muscle recovery, as measured by peak torque or maximal muscle force, also recovered much quicker in the massaged muscles than in the unmassaged muscles [47,55]. As with post-damage muscle inflammatory response, there was also a dose-dependent effect of MLL in limiting muscle damage and enhancing the return of muscle strength, with higher magnitudes and frequencies of MLL force application resulting in significantly better outcomes [59]. Peak torque and muscle strength as measures of post-damage muscle recovery are also used in human studies on massage and will be discussed in that context in the subsequent subsection.

Other studies have also reported similar findings. One study [5] applied manual application of massage-like skin rolling and manipulation three times per week on rat forearm muscles following repetitive reaching/pulling tasks for two hours per day for 12 weeks. Controls received no intervention. As well as the previously noted reduction in muscle inflammation, the massaged muscles and tendons had significantly less fibrosis and histological evidence of muscle damage and disruption. Notably, forearm muscle grip strength was much better maintained in the massaged animals than in the controls, indicating less muscle damage and disruption induced by the exercise protocol [5].

Seo et al. [61] also reported quicker muscle repair, myogenesis, satellite cell activation and return of muscle strength in rodent muscles severely injured by the injection of toxin when the muscles were exposed to robotic massage-like manipulation in conjunction with mechanical loading following the damage. These benefits were linked to a significant reduction in inflammation and neutrophil infiltration following the damage.

Liu et al. [67] applied regulated manual massage pressure to rat muscles injured through downhill running and noted significantly less muscle structural damage, an amelioration of the loss of muscle force, and enhancements to integrin and the muscle basement membrane related to improved protection from muscle damage. It has been suggested that mitogen-activated protein kinase (MAPK) and other related signaling pathways mediate these responses [68]; however, this has yet to be fully confirmed with experimental data.

Recent studies examining rodent muscles during recovery from disuse atrophy have demonstrated that the repeated application of MLL will enhance muscle protein synthesis and the maintenance of ribosomal integrity and may stimulate muscle satellite cell proliferation, leading to an overall enhancement of muscle recovery when performed in addition to muscle loading [6,66,72]. Increases in muscle satellite cell activation and number as assessed by Pax-7 immunohistochemistry were also seen following MLL in unexercised young (10 months) and old (30 months) rats [73]. While further confirmational research is still needed, there is evidence for positive effects of massage on muscle satellite cell numbers in animal models.

These positive effects of MLL on factors affecting muscle repair were not effective in enhancing overall net protein synthesis during unloaded disuse muscle atrophy [6,66]. Interestingly, protein synthesis and muscle satellite cell activation were also enhanced by MLL in the non-massaged limb [66,72]. It is possible that MLL stimulates protein synthesis and muscle satellite cell activation in the contralateral limb muscles via stimulation of the release of myokine and exosome signaling factors from the massaged muscle into the circulation or through neural stimulation via the sympathetic nervous system [66,73,74]. Sex differences in massage effects were also observed, with significantly diminished responses seen in female relative to male rats [6,66]. Although physiologically different from exercise-induced muscle injury, studies using MLL with recovery from muscle atrophy could still speak to common mechanisms of massage-like interventions in overall muscle repair.

Older rodents also did not exhibit as robust a response to MLL during and in recovery from muscle atrophy as younger male rats [75]. This lack of response was also not enhanced by a greater MLL force loading in older rats, and, unlike that seen in younger rats, greater MLL in recovery from muscle atrophy actually resulted in more muscle damage [76].

Animal models have consistently demonstrated positive effects of massage-like interventions in diminishing post-contraction indices of muscle damage and enhancing the recovery of post-damage muscle force, muscle structural integrity and resistance to further injury [48,67]. These effects of massage-like interventions are linked in part to the ability of massage-like interventions to limit post-damage muscle inflammatory responses and the infiltration of pro-inflammatory leukocytes, and also to possibly act via enhanced muscle protein synthesis, satellite cell activation and the enhancement of muscle ultrastructural integrity. These benefits of massage are influenced by the timing and variability in force and duration of the massage-like application. The sex and age of the animals are also factors in the effectiveness of massage-like interventions in enhancing muscle recovery from unloading-induced muscle atrophy.

### 8.3. Massage Influence on Indices of Muscle Recovery in Humans

Unlike the ubiquitously positive effects of massage-like actions on post-damage muscle repair and functional and force recovery in animal models, the documented effects of massage on the recovery of exercise- or otherwise-damaged muscle in humans are more equivocal. The research findings on massage effects in animals may not necessarily directly mimic human responses due to physiological differences. Nevertheless, sufficient similarities likely exist such that at least some of the potential physiological benefits of massage that have been demonstrated in animals could be translatable to humans.

The loss of muscle force-generating capacity is a consequence of muscle damage, and its recovery follows a similar timeline to the repair of muscle contractile, membrane and structural components [20]. The loss of muscle force following damage can be attributed to the disruption of muscle sarcomeres, muscle t-tubules and membranes and the associated excitation–contraction uncoupling and disruption of calcium channels and calcium signaling [53,71]. The repair and remodeling of muscle contractile elements, membranes and infrastructure during post-damage muscle recovery are directly reflected in the rate of return of muscle force [20,72]. Measures of voluntary maximal muscle isometric, isokinetic or dynamic contraction force have been commonly used in human studies to indirectly track muscle repair and recovery, and this measure has been noted as being the most accurate and indicative non-invasive measure of the rate of post-damage muscle repair [20,44,77].

Numerous systematic reviews and controlled studies on the effectiveness of massage therapy on recovery from exercise-induced muscle damage have found little consistent evidence for massage of any type to enhance muscle repair, as determined by the rate of muscle force recovery for up to a week following damaging exercise [3,11,13,25,29,44,78]. These results are relatively consistent whether the application of massage begins soon after the completion of the muscle disrupting exercise, is delayed until 24 h after exercise, is applied only once or repeated daily for up to a week and/or is varied in duration and intensity [3,13,14,44]. Dynamic measures of sport performance or recovery from exercise in athletic populations have, with some variability, also not generally been reported to be significantly improved by the application of massage interventions with various timings, intensities and durations [22,30,79].

The leakage of muscle enzymes such as creatine kinase (CK) into the circulation is an indirect indicator of muscle membrane disruption following damaging exercise [53]. While not quantitatively indicative of overall muscle and specific myofibular damage and repair, blood CK levels have been used as surrogate, semi-quantitative indicators of post-exercise muscle membrane disruption and recovery [53]. While, not completely consistent, massage has generally been shown to reduce the post-muscle damage levels of creatine kinase to varying degrees up to 72 h post-exercise in a number of human studies [29,36,44,52,80].

Post-exercise, muscle membrane disruption and related inflammatory responses may be factors in stimulating muscle repair [8]. Whether this is a mechanism by which massage may potentially affect post-exercise muscle recovery is unknown. The application of MLL to unexercised older and younger rodents did not demonstrate any effects of massage on histochemically determined membrane disruption [73]. Nevertheless, massage may potentially have some positive effects on limiting muscle membrane damage and perhaps enhancing muscle membrane repair following exercise-induced damage in humans, possibly via some of the mechanotransduction-related mechanisms previously noted. These possibilities require further research.

There is currently no data from humans on the effects of massage of any type on the other indices of muscle recovery seen in animal models, such as changes to protein synthesis or degradation rates, muscle satellite cell activation or their potential signaling pathways.

### 8.4. Reconciling Inconsistencies in Massage Efficacy Between Animal and Human Based Studies

In light of the positive effects of massage-like interventions in controlled animal studies of post-damage muscle recovery, the lack of positive results as determined by the rate of muscle force recovery in humans is puzzling. In addition to direct evidence from animal studies for reduced inflammatory markers and the enhanced rate of muscle satellite cell activation, protein synthesis, structural and membrane repair and reduced muscle fibrosis with MLL and other massage-like interventions, animal studies have also correlated these changes with an enhanced recovery of muscle force [4,5]. Hence, the return of muscle force following muscle damage can be seen as a reliable non-invasive indicator of muscle recovery in these animal models. The lack of consistent benefits of massage on the rate of muscle force recovery following muscle damage in humans would suggest the lack of benefit of a range of massage applications, timings and techniques on muscle repair.

One possible explanation for the conflicting results in the efficacy of massage for enhancing muscle repair in human and animal studies may be attributed to the lack of consistency of massage techniques, massaging pressures, timing and durations in human studies [12]. As noted in the review of the effectiveness of massage-like interventions on post-damage muscle inflammatory responses and the rate of muscle repair in animal models, there were relatively narrow windows for timing, massage pressure and duration, as well as possible roles of age and sex of the animals, on the effectiveness of massage [4,76]. If only very specific massage forces, techniques, durations or timings of massage interventions may be effective and are not yet empirically documented in humans, the wide range of massage techniques used over the course of human massage research would likely not have consistently used the narrow window of potentially successful massage techniques needed to induce positive effects.

Some human studies have also had limited numbers of subjects and could in some cases have failed to demonstrate positive effects due to a lack of sufficient statistical power [2,29,34]. A lack of adequate controls and sham conditions and possible placebo effects have also limited the validity of some human studies on massage [34,44].

Additionally, potential differences in the physiology of human and animal muscles, their responses, and recovery mechanisms, as well as human and animal differences in muscle damage induction and measurements of muscle repair and recovery, could in part account for some of the above discrepancies [47,77].

It may also be that, as in animal models, massage intervention in humans may only be efficacious if applied immediately following muscle damage, using a specific and yet undefined duration and intensity and specific and yet undefined techniques tailored to the age and sex of the subjects. As previously noted, the aim of massage is likely to enhance the signaling of muscle protein synthesis and repair pathways and dampen muscle inflammatory signaling by mechanotransduction-stimulated pathways [4,55,59,60,61]. The potentially narrow window of the positive effectiveness of massage techniques and timings, while evident and defined in animal models, has yet to be demonstrated or documented in human studies. It seems possible that only a very limited range of timings, techniques, pressure applications and other massage variables may ultimately prove effective in positively enhancing mechanotransduction-induced pathways to improve muscle recovery and repair. Until these parameters are researched and defined, massage therapists are essentially working blind, and massage therapy, with its wide range of possible therapist-determined and widely disseminated techniques, most of which lack a strong empirical and evidence-based foundation for their use, will in most cases prove, as demonstrated by the current research findings, to be largely ineffective in enhancing muscle recovery from exercise-induced muscle damage.

## 9. Summary, Conclusions and Ways Forward

The following are the main findings and recommendations of this review:

1. Except for modest positive effects on DOMS sensation, blood CK levels and inflammatory markers, various forms of massage have not been consistently demonstrated to enhance acute or longer-term indicators of recovery from exercise-induced muscle damage in humans. No consistent benefits of massage have been shown in reducing muscle edema or swelling or the recovery of post-damage muscle force. Muscle blood flow is also unaffected by massage. Practitioners should be aware of these current limitations and parameters of evidence for massage effectiveness in humans.
2. In contrast to studies in humans, massage-like interventions in animal models have consistently shown significant benefits in reducing post-damage indices of muscle disruption and inflammation and in enhancing muscle repair, protein synthesis and the recovery of muscle force. These animal models have demonstrated that only very specific timing, force, duration and technique in the massage application will be effective in enhancing muscle repair and recovery. The effectiveness of massage on muscle recovery will also be influenced by the age and sex of the animal and may need to vary in technique, force and timing for optimal effects relative to age and sex.
3. Consistent evidence supporting massage’s effects on diminished post-exercise leukocyte infiltration of muscles, as well as effects on protein synthesis and degradation, possibly because of massage-induced mechanotransduction effects, may demonstrate the means by which massage affects post-damage muscle recovery. Exact signaling pathways and systemic inflammatory mediators require further experimental confirmation.
4. The perhaps very narrow “window of effectiveness” for massage timing, technique, force and duration has yet to be defined in humans, and the lack of demonstrated effectiveness of massage in influencing most aspects of post-damage muscle recovery may be due to the wide variety and inconsistency of massage forms and variables and experimental limitations that have been applied during massage and muscle repair research in humans. One effective massage protocol reported in animals involves an immediate post-damage roller massage of “modest” pressure for 30 min repeated daily for 4 days. It is not yet been confirmed whether this timing, duration or frequency of massage would be effective in humans, nor has an optimal pressure of massage application been determined for various human populations.
5. To address this, systematic research specifically defining and testing a variety of massage applications related to timing, force, technique, duration and other variables in robust experimental designs needs to be conducted in order to help determine what, if any, specific massage applications and forms might be most effective in enhancing post-damage muscle repair in humans as well as their physiological mechanisms, which may differ from animal models. Until such data are forthcoming, the application of massage to enhance muscle repair in humans will continue to be performed without empirical evidence and likely with very limited success.

## Data Availability

No new data were created or analyzed in this study.

## Abbreviations

The following abbreviations are used in this manuscript:

AKT	Protein Kinase b
CK	Creatine Kinase
DNIC	Diffuse Noxious Inhibitory Control
DOMS	Delayed Onset Muscle Soreness
ECM	Extracellular Matrix
EMG	Electromyography
IGF-1	Insulin-Like Growth Factor-1
IL	Interleukin
MAPK	Mitogen-Activated Protein Kinase
MLL	Massage-Like Compressive Loading
mTor	Mechanistic Target of Rapamycin
TGF-β	Transforming Growth Factor-beta
TNF-α	Tumor Necrosis Factor-alpha
